# Associations Between the Horizontal Force‐Velocity Profile and Change of Direction Asymmetries and Phase‐Specific Performance in Elite U21 Football Players

**DOI:** 10.1002/ejsc.70219

**Published:** 2026-07-09

**Authors:** Adam Lipčák, Ana Carolina Paludo, Tomáš Kalina, Dušana Augustovičová, Aris Schnelldorfer, Radovan Hadža, Michal Hrubý

**Affiliations:** ^1^ Faculty of Sports Studies Masaryk University Brno Czech Republic; ^2^ Department of Psychology University of Cyprus Nicosia Cyprus; ^3^ Faculty of Physical Education and Sport Comenius University Bratislava Slovakia

**Keywords:** diagnostics, FVP, limb dominance, soccer, sprint

## Abstract

The horizontal force–velocity profile (H‐FVP) provides insights into sprint mechanical capacities, but its relationship with change of direction (COD) performance, inter‐limb asymmetry, and phase‐specific outcomes in elite football remains unclear. This study examined associations between H‐FVP variables and COD performance in 15 elite male players from the Czech Republic U21 national team. Athletes performed a 40 m sprint and a 15‐0‐5 COD test with a motorized resistance device. H‐FVP variables (F0, V0, Pmax, RF, DRF) were derived from sprint trials, while COD outcomes included overall times, asymmetry indices, and specific phases. Correlations were calculated between sprint mechanics and COD outcomes. Significant relationships were observed between H‐FVP and COD performance on the dominant leg. Faster COD times were correlated with higher F0 (*r* = −0.73, *p* < 0.01), Pmax and RF (*r* = −0.72, *p* < 0.01), and with lower DRF. Sprint performance also correlated negatively with F0 and Pmax. In contrast, relationships between H‐FVP and COD asymmetry were limited, although V0 showed positive correlations with overall and Phase 1b asymmetry. Phase‐specific analysis revealed stronger links with Phase 1a and initial acceleration, particularly on the dominant side, whereas deceleration and re‐acceleration phases showed weaker or inconsistent correlations. These findings indicate that H‐FVP, especially F0 and Pmax, is strongly related to sprint and early COD performance but provides limited information on inter‐limb asymmetry and later COD phases. Integrating horizontal sprint profiling with phase‐specific COD assessments may therefore enhance athlete monitoring and training design in elite football players.

## Highlights

- Horizontal force–velocity profile variables, particularly maximal force and power, are strongly related to sprint and early change‐of‐direction performance in elite football players.
- Relationships between sprint mechanical properties and inter‐limb asymmetries are limited, with velocity showing the only consistent associations.
- Phase‐specific analysis revealed that sprint mechanical outputs mainly influence the initial acceleration phase, while deceleration and re‐acceleration depend on other physical qualities.

## 1 Introduction

High‐intensity locomotor actions, including acceleration, sprinting, and change of direction (COD), occur frequently during football matches and contribute to decisive moments such as creating space, winning duels, and generating or preventing scoring opportunities (Barnes et al. 2014; Faude et al. 2012). Moreover, rapid deceleration and COD manoeuvres impose substantial mechanical demands on the lower limbs (Nimphius et al. 2018), while previous injury has been identified as an important risk factor for subsequent hamstring and knee injuries in football players (Arnason et al. 2004). Therefore, understanding the mechanical and neuromuscular determinants of sprinting and COD performance is a central objective for football performance and injury‐prevention research (Arnason et al. 2004).

One approach that has gained attention in recent years is the assessment of the horizontal force–velocity profile (H‐FVP). The H‐FVP provides information on how athletes generate and apply force throughout sprint acceleration and is commonly described by key parameters such as theoretical maximal force (F0), maximal velocity (V0), maximal power output (Pmax), the ratio of horizontal‐to‐resultant force (RF), and the decrement in RF with increasing velocity (DRF) (Samozino et al. 2022). Profiling these variables offers valuable insights into an athlete's sprint mechanical capacities and can be used to guide individualized training interventions aimed at improving acceleration and sprint performance (Jimenez‐Reyes et al. 2018; Morin and Samozino 2016).

**FIGURE 1:**
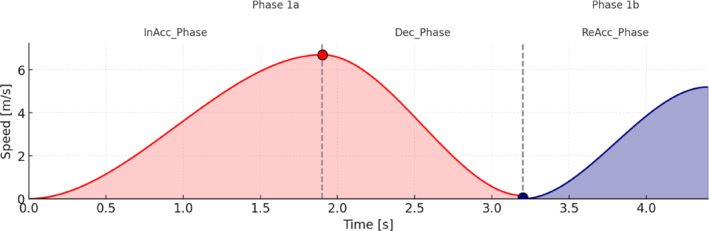
Phase‐specific segmentation of the 15‐0‐5 COD test. Dec = deceleration; InAcc = initial acceleration; m/s = meters per second; ReAcc = re‐acceleration; s = second.

COD ability, however, is more complex than linear sprinting because it requires the coordinated execution of rapid deceleration, redirection of whole‐body momentum, and subsequent re‐acceleration (Nimphius et al. 2018; Westheim et al. 2023). Several tests have been developed to assess COD ability, including the 505 test, pro‐agility shuttle, *T*‐test, and variations such as the 15‐0‐5 test (Kozinc and Sarabon 2022; Nimphius et al. 2018). Beyond overall COD performance times, recent research has emphasized the importance of examining COD phases separately (Buchheit and Eriksrud 2024; Westheim et al. 2023). Distinct phases such as Phase 1a and Phase 1b (often divided into sub‐phases like initial acceleration, deceleration, turn execution and re‐acceleration, see Figure 1) are supported by different mechanical and neuromuscular demands. Phase‐specific analyses may therefore provide a more detailed understanding of the determinants of COD performance than overall time alone (Westheim et al. 2023).

While previous studies have shown associations between sprint mechanical outputs and COD performance (Baena‐Raya et al. 2021; Sánchez‐López et al. 2023), much less is known about the relationship between H‐FVP and inter‐limb asymmetry as well as phase‐specific COD outcomes. Inter‐limb asymmetries have been linked to both performance decrements (Coratella et al. 2018) and increased injury risk (Beato et al. 2021), but evidence regarding their interaction with sprint mechanical variables remains inconsistent. Furthermore, the extent to which horizontal sprint mechanics explain performance in distinct COD phases has not been fully established, particularly in the professional football population.

Therefore, the aim of this study was to examine the associations between horizontal force–velocity profile variables and COD performance in elite U21 football players, with specific attention to inter‐limb asymmetries and phase‐specific outcomes.

## 2 Materials and Methods

### 2.1 Experimental Approach

This cross‐sectional study was conducted during a training camp of the U21 national team. A 40‐m linear sprint was used to derive H‐FVP variables in accordance with established sprint‐profiling procedures (Morin and Samozino 2016), whereas a 15‐0‐5 COD test performed with a motorized resistance device was used to obtain bilateral and phase‐specific performance outcomes, from which inter‐limb differences were calculated (Westheim et al. 2023). All testing was performed under standardized conditions with consistent equipment settings, surface type, and environmental factors. The methodological design allowed for the collection of sprint mechanical variables alongside COD performance measures within a single, controlled session.

### 2.2 Participants

Fifteen elite male football players representing the Czech Republic U21 national team participated in the study. Inclusion criteria were: (i) membership in the Czech Republic U21 national team, (ii) being free from musculoskeletal injury at the time of testing, and (iii) completion of at least six months of continuous football training prior to the assessments. Exclusion criteria were: (i) any current or recent injury (within the previous 3 months) that could affect performance, (ii) incomplete participation in the testing session. Written informed consent was obtained from all participants, and ethical approval was granted by the institutional ethics committee. In addition, the data collection was conducted as part of a formal agreement between a national football federation and a collaborating university, with the primary purpose of academic reporting on athlete performance.

### 2.3 Experimental Design

All testing was conducted during a single session. Before the assessments, players completed a standardized warm‐up based on the RAMP protocol (Raise, Activate, Mobilize and Potentiate), including dynamic mobility drills, progressive acceleration runs, and short sprints to ensure optimal neuromuscular readiness (Jeffreys 2007). Following the warm‐up, athletes performed two field‐based tests using the 1080 Sprint device (1080 Motion AB, Lidingö, Sweden). First, a 40‐m linear sprint with constant resistance was executed to collect data for H‐FVP. Subsequently, players completed a 15‐0‐5 COD test, which includes a 10 m run‐up before performing the 5 m COD segment. This offers a more comprehensive assessment of specific phase performance of COD and inter‐limb differences compared to the traditional 5‐0‐5 test (Buchheit and Eriksrud 2024; Westheim et al. 2023). Surface conditions, footwear, and equipment settings were identical for all players, and the testing was supervised by experienced strength and conditioning staff to ensure consistent application of the protocol.

#### 2.3.1 Linear Sprint Over 40 m

The H‐FVP was assessed using a 40‐m linear sprint with the 1080 Sprint device. Players started from a standing split stance (two‐point standing start) with a tethered waist belt connected to the device. The resistance was set to 3 kg in “No Fly Weight” mode, as recommended for valid H‐FVP (1080 Motion 2025a). Each player performed two maximal effort trials. The trial automatically classified by the device as “confidence” was used for further analysis (1080 Motion 2025b). If both trials met the confidence criteria, the faster attempt was selected, whereas in cases where neither trial was considered valid, a third attempt was provided. A minimum of 3 min of passive recovery was allowed between trials. The H‐FVP was computed by the 1080 Sprint software, which estimates mechanical parameters of sprint performance (F0, V0, Pmax, RF, DRF) based on the Samozino–Morin approach. This method has been shown to be a reliable and valid procedure for profiling horizontal sprint mechanics (Morin and Samozino 2016).

#### 2.3.2 Change of Direction Test (15‐0‐5)

The COD test was performed using the 15‐0‐5 protocol with the 1080 Sprint device. The resistance was consistently set to 3 kg in “No Fly Weight” mode. Players wore a tethered waist belt connected to the device throughout the entire movement. From a standing split stance, athletes accelerated for 15 m toward the device (assisted direction), executed a 180° turn, and sprinted 5 m away from the device (resisted direction). Each player completed two turns on the dominant (D) leg and two turns on the non‐dominant (ND) leg. The best trial was retained for analysis, with a minimum of 2 min of passive recovery provided between attempts.

Asymmetry in COD performance was quantified between dominant and non‐dominant legs for overall completion time, as well as for specific COD phases and sub‐phases (Phase 1a and Phase 1b initial acceleration, deceleration and re‐acceleration). Asymmetry was calculated using the formula (Dos’Santos et al. 2017):

$$\mathrm{Asymmetry}\;\left(\%\right)=\frac{D-\text{ND}}{D}\cdot 100$$

In addition to asymmetry analysis, performance within individual COD phases was examined. Phases and sub‐phase metrics were derived directly from the 1080 Sprint system, including COD Phase 1a, COD Phase 1b, initial acceleration, deceleration, and re‐acceleration phases, with values reported separately for the dominant and non‐dominant limbs (Figure 1).

### 2.4 Data Analysis

All statistical analyses were performed using Python 3.11 (Python Software Foundation, Wilmington, DE, USA) with the pandas, SciPy, Matplotlib, and Seaborn libraries. Continuous variables were summarised as mean ± standard deviation (SD), together with minimum and maximum values. The normality of each variable was assessed using the Shapiro–Wilk test and visual inspection of the data distributions.

Associations between H‐FVP variables and COD outcomes were examined in three groups: (i) overall COD and linear sprint performance, (ii) inter‐limb asymmetry indices, and (iii) phase‐specific COD outcomes. Pearson's correlation coefficient was used when the assumptions of normality and linearity were satisfied. Spearman's rank correlation coefficient was used for non‐normally distributed variables or when these assumptions were not met. All statistical tests were two‐sided, and statistical significance was accepted at *p* < 0.05.

Correlation coefficients were interpreted based on their absolute magnitude as negligible (< 0.10), weak (0.10–0.39), moderate (0.40–0.69), strong (0.70–0.89), or very strong (≥ 0.90) (Schober et al. 2018). Correlation results were reported using the respective correlation coefficient and exact *p* value. Given the exploratory nature of the study and the small sample size, the magnitude of the correlation coefficients was considered alongside statistical significance.

Correlation matrices were visualised as heatmaps using a diverging color scale ranging from blue through white to red. Blue cells indicate negative correlations, red cells indicate positive correlations, and cells approaching white indicate correlations close to zero. Increasing color intensity represents a greater absolute magnitude of the correlation coefficient. Asterisks indicate statistical significance (* *p* < 0.05; ** *p* < 0.01).

## 3 Results

### 3.1 Descriptive Statistics

Descriptive statistics for all measured variables are summarized in Table 1. The table reports mean, standard deviation, minimum, and maximum values for H‐FVP parameters (F0, V0, Pmax, RF, DRF) as well as COD test outcomes. COD performance was analyzed for both dominant and non‐dominant legs, including overall completion times, asymmetry indices, and phase‐specific measures (Phase 1a, Phase 1b, initial acceleration, deceleration, and re‐acceleration).

**TABLE 1:** Descriptive performance variables of U‐21 soccer players (*n* = 15).

Variables	Mean	SD	Min	Max
COD performance [s] D	4.37	0.24	4.06	4.96
COD performance [s] ND	4.41	0.16	4.19	4.75
COD overall diff. [%]	3.74	3.8	0.45	15.01
COD phase 1a D	3.13	0.18	2.89	3.57
COD phase 1a ND	3.17	0.14	2.97	3.41
COD diff. phase 1a	4.04	3.73	0	14.83
COD phase 1b D	1.24	0.07	1.09	1.39
COD phase 1b ND	1.24	0.06	1.14	1.42
COD diff. phase 1b	5.95	4.45	0.81	15.45
InAcc_Phase D	1.85	0.16	1.57	2.27
InAcc_Phase ND	1.88	0.14	1.71	2.17
Dec_Phase D	1.27	0.1	1.13	1.49
Dec_Phase ND	1.29	0.1	1.18	1.44
ReAcc_Phase D	1.24	0.07	1.09	1.39
ReAcc_Phase ND	1.24	0.06	1.14	1.42
Sprint performance [s]	5.87	0.21	5.46	6.26
F0 [N/kg]	7.59	0.62	6.08	8.71
V0 [m/s]	9.38	0.44	8.72	10.5
Pmax [W/kg]	17.77	1.51	15.97	21.27
RF [%]	50.32	2.53	44.1	54.9
DRF [%]	−6.72	0.6	−7.3	−4.87

Abbreviations: COD = change of direction; D = dominant; Dec = deceleration; diff. = difference; DRF = decrease in the ratio of horizontal to resultant force; F0 = theoretical maximal force; InAcc = initial acceleration; m/s = meters per second; Max = maximum; Min = minimum; N/kg = newtons per kilogram; ND = non‐dominant; Pmax = maximal power; ReAcc = re‐acceleration; RF = ratio of horizontal to resultant force; s = second; SD = standard deviation; V0 = theoretical maximal velocity; W/kg = watts per kilogram.

### 3.2 Correlations Between H‐FVP and Overall Performance

**FIGURE 2:**
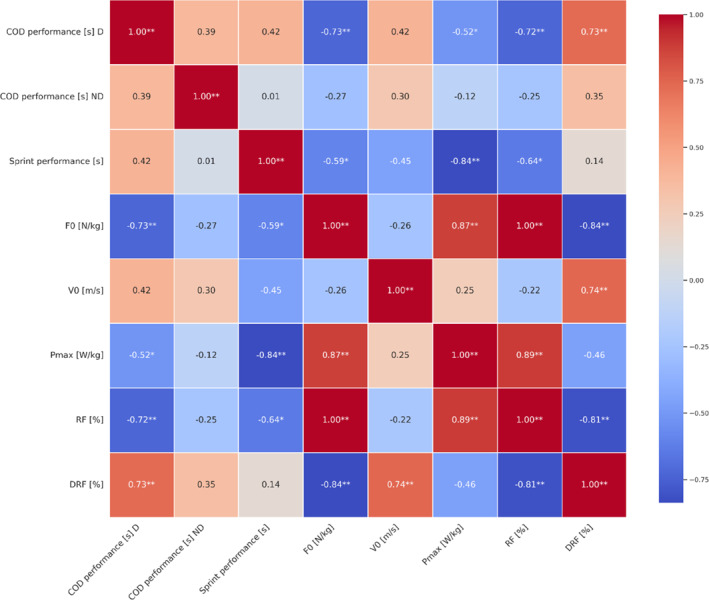
Correlation matrix between H‐FVP and overall performance outcomes. Cell values represent correlation coefficients. Blue indicates negative correlations, red indicates positive correlations, and increasing color intensity represents a greater absolute magnitude of the correlation; * = *p* < 0.05; ** = *p* < 0.01; D = dominant; DRF = decrease in the ratio of horizontal to resultant force; F0 = theoretical maximal force; m/s = meters per second; N/kg = newtons per kilogram; ND = non‐dominant; Pmax = maximal power; RF = ratio of horizontal to resultant force; s = second; V0 = theoretical maximal velocity; W/kg = watts per kilogram.

Correlation coefficients between H‐FVP variables and overall performance outcomes (COD and linear sprint) are presented in Figure 2. Significant correlations were observed between H‐FVP parameters and COD performance on the dominant leg. COD time (dominant) showed strong negative correlations with F0 (*r* = −0.73, *p* < 0.01), Pmax (*r* = −0.52, *p* < 0.05), and RF (*r* = −0.72, *p* < 0.01), while a positive correlation was identified with DRF (*r* = 0.73, *p* < 0.01). No significant correlation was found between COD time on the non‐dominant leg and H‐FVP variables. Sprint performance was significantly related to sprint mechanical outputs (F0 and Pmax). Sprint time was negatively correlated with F0 (*r* = −0.59, *p* < 0.05) and Pmax (*r* = −0.84, *p* < 0.01). Athletes with higher F0 and Pmax values demonstrated superior performance in both linear sprinting and COD tasks on the dominant leg.

### 3.3 Correlations Between H‐FVP and COD Asymmetries

**FIGURE 3:**
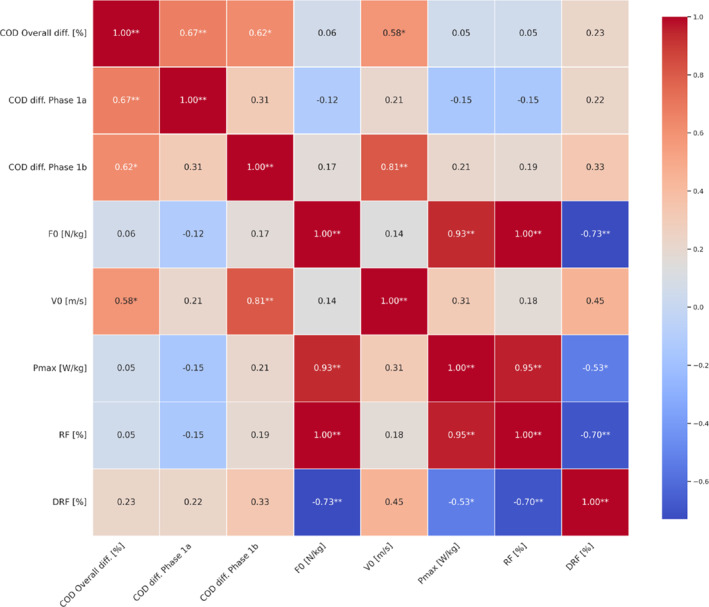
Correlation matrix between H‐FVP and COD asymmetries. Cell values represent correlation coefficients. Blue indicates negative correlations, red indicates positive correlations, and increasing color intensity represents a greater absolute magnitude of the correlation; * = *p* < 0.05; ** = *p* < 0.01; COD = change of direction; diff. = difference; DRF = decrease in the ratio of horizontal to resultant force; F0 = theoretical maximal force; m/s = meters per second; N/kg = newtons per kilogram; Pmax = maximal power; RF = ratio of horizontal to resultant force; V0 = theoretical maximal velocity; W/kg = watts per kilogram.

Correlations between H‐FVP variables and COD asymmetry indices are shown in Figure 3. COD overall asymmetry was positively associated with V0 (*r* = 0.58, *p* < 0.05), but no significant relationships were observed with F0, Pmax, RF, or DRF. Phase‐specific asymmetries showed partly divergent patterns. COD Phase 1a asymmetry showed a moderate correlation with overall asymmetry (*r* = 0.67, *p* < 0.01). However, it was not significantly correlated with any of the FVP parameters. By contrast, COD Phase 1b asymmetry was strongly correlated with V0 (*r* = 0.81, *p* < 0.01), while again showing no meaningful associations with other FVP outputs. Sprint mechanical parameters showed weak correlations with inter‐limb asymmetries in COD performance. Velocity‐related measures (V0) were the exception, demonstrating consistent correlations with both overall and phase‐specific asymmetry.

### 3.4 Correlations Between H‐FVP and COD Specific Phases

**FIGURE 4:**
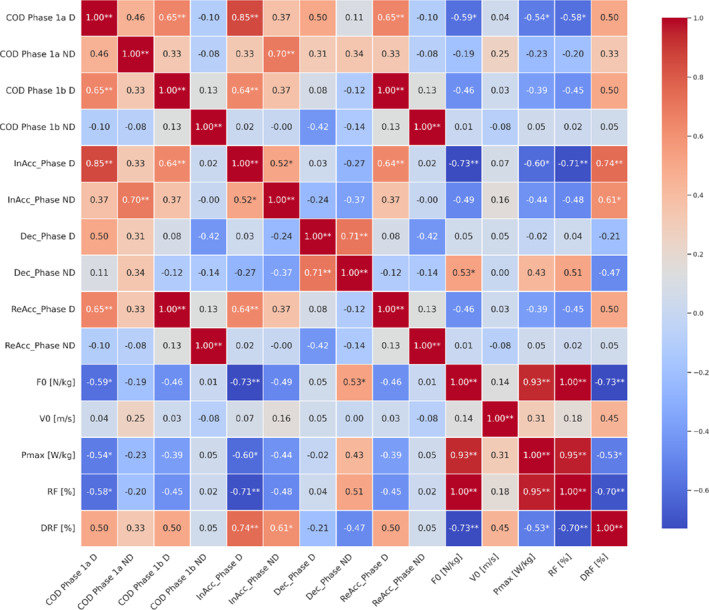
Correlation matrix between H‐FVP and COD specific‐phases. Cell values represent correlation coefficients. Blue indicates negative correlations, red indicates positive correlations, and increasing color intensity represents a greater absolute magnitude of the correlation; * = *p* < 0.05; ** = *p* < 0.01; COD = change of direction; D = dominant; Dec = deceleration; DRF = decrease in the ratio of horizontal to resultant force; F0 = theoretical maximal force; InnAcc = initial acceleration; m/s = meters per second; N/kg = newtons per kilogram; ND = non‐dominant; Pmax = maximal power; ReAcc = re‐acceleration; RF = ratio of horizontal to resultant force; V0 = theoretical maximal velocity; W/kg = watts per kilogram.

Correlations between H‐FVP variables and COD specific phases are shown in Figure 4. Significant correlations were found primarily between F0, Pmax, RF, and DRF with different COD specific phase measures. COD Phase 1a (dominant leg) correlated negatively with F0 (*r* = −0.59, *p* < 0.05), Pmax (*r* = −0.54, *p* < 0.05), and RF (*r* = −0.58, *p* < 0.05). Similar patterns were observed for InAcc_Phase on the dominant side, which was negatively associated with F0 (*r* = −0.73, *p* < 0.01), RF (*r* = −0.71, *p* < 0.01), and positively with DRF (*r* = 0.74, *p* < 0.01). In contrast, sub‐phases on the non‐dominant side exhibited fewer and weaker relationships. For example, InAcc_Phase ND was moderately correlated with DRF (*r* = 0.61, *p* < 0.05), whereas deceleration and re‐acceleration sub‐phases showed no consistent associations with FVP outputs. Sprint mechanical properties derived from the H‐FVP showed the strongest correlations with Phase 1a (initial acceleration), particularly on the dominant limb. Correlations with later sub‐phases, including deceleration and re‐acceleration, were weaker.

## 4 Discussion

The main finding of the present study was that H‐FVP parameters were significantly correlated with overall COD performance and specific COD phases, whereas their relationships with inter‐limb asymmetries were limited. More specifically, greater F0, Pmax, and RF were linked to faster COD times and superior execution of Phase 1a and initial acceleration, particularly on the dominant limb. In contrast, weaker and less consistent correlations were observed between H‐FVP outputs and Phase 1b or re‐acceleration performance, as well as with COD asymmetry indices. These results suggest that horizontal sprint mechanics predominantly influence the initial part of COD execution, while other qualities, such as eccentric strength, braking capacity, and technical skill, may play a more prominent role in later phases of directional changes.

### 4.1 FVP and Overall Performance (COD and Linear Sprint)

The present study demonstrated that H‐FVP variables were strongly associated with overall COD and linear sprint performance, particularly on the dominant limb. Greater F0, Pmax, and RF were linked to shorter COD times, whereas DRF showed positive associations, indicating that players with superior horizontal force and power capacities were more effective in executing directional changes. Similarly, sprint times were negatively correlated with F0 and Pmax, confirming the relevance of sprint mechanical outputs to maximal sprinting ability.

These results are consistent with previous research highlighting the importance of horizontal mechanical capacities for acceleration and sprint performance (Jimenez‐Reyes et al. 2018; Morin and Samozino 2016). Sánchez‐López et al. (2023) also reported strong associations between horizontal power production and COD ability in team‐sport athletes, suggesting that sprint mechanics may support agility‐related tasks. In agreement with these findings, Zhang et al. (2024) recently demonstrated that V0, RF, and FVslope were significant determinants of COD performance, further supporting the relevance of sprint mechanical profiling for explaining inter‐individual differences in agility‐related tasks. At the same time, our findings align with the notion that linear sprint and COD share common mechanical determinants but remain distinct skills (Nimphius et al. 2016).

From a practical standpoint, these findings support the integration of horizontal sprint profiling into athlete monitoring, as improvements in horizontal force and power production capacities are likely to translate into both faster linear sprinting and enhanced COD performance.

### 4.2 FVP and Asymmetries

In the present study, H‐FVP variables were only weakly associated with COD asymmetry indices, with the exception of V0, which showed significant positive correlations with both overall COD asymmetry and Phase 1b (re‐acceleration) asymmetry. This suggests that athletes with greater sprint velocity capacity may also express larger inter‐limb discrepancies when re‐accelerating after a directional change, highlighting the complexity of the relationship between linear sprint mechanics and unilateral COD performance.

These findings are in line with recent evidence showing that inter‐limb asymmetries have only a limited and inconsistent impact on sprint and COD performance (Exell et al. 2017; Fox et al. 2023). Exell et al. (2017) demonstrated that maximal‐velocity sprinting asymmetries exist even in elite sprinters but do not necessarily constrain performance. Similarly, Fox et al. (2023) concluded in a meta‐analysis that asymmetry–performance associations are generally small (*r* = 0.20 – 0.24), supporting the notion that asymmetry alone is not a strong predictor of COD or sprint outcomes. Our data extend these findings by indicating that H‐FVP, despite being a powerful determinant of sprint and COD performance, does not directly explain the asymmetry observed between dominant and non‐dominant limbs. This partly aligns with the results of Zhang et al. (2024), who also identified V0 as an important determinant of COD asymmetry. However, unlike our findings, they additionally reported significant contributions of DRF and FVslope. Such discrepancies may be related to differences in sample characteristics or sample size, which could influence the detection of smaller effects.

Recent applied studies have also suggested that asymmetry profiles are influenced by multiple factors beyond sprint mechanics, including neuromuscular control, movement coordination, and training history (Baena‐Raya et al. 2021; DosʼSantos et al. 2019). The positive link between V0 and Phase 1b asymmetry in our cohort may reflect the fact that players with greater top‐end velocity are able to exploit their dominant limb more effectively during re‐acceleration, thereby amplifying inter‐limb differences. From a practical standpoint, this underlines the importance of incorporating unilateral COD and eccentric strength drills aimed at reducing performance discrepancies between limbs, which may not only improve COD efficiency but could also contribute to lowering injury risk.

### 4.3 FVP and COD Specific Phases

Our results demonstrated that H‐FVP parameters were most strongly associated with COD Phase 1a and initial acceleration, particularly on the dominant limb, while relationships with deceleration and re‐acceleration sub‐phases were weaker or inconsistent. Specifically, higher F0, Pmax, and RF (and lower DRF) were linked to superior performance in the early phases of COD, whereas later phases did not display consistent associations with sprint mechanical outputs. This indicates that sprint‐derived mechanical capacities primarily support the ability to generate horizontal force and velocity at the onset of COD but are less influential once athletes must decelerate, turn, and re‐accelerate.

These findings are consistent with previous work showing that acceleration‐related phases of COD are most dependent on horizontal force and power production (Nimphius et al. 2016; Sánchez‐López et al. 2023), whereas braking and re‐acceleration rely more on eccentric strength and technical execution (Baena‐Raya et al. 2021; DosʼSantos et al. 2019). Importantly, the methodological framework of our study is supported by recent evidence confirming the reliability of phase‐specific outcome measures derived from motorized resistance devices such as the 1080 Sprint (Westheim et al. 2023). This reinforces the validity of our phase‐level observations, suggesting that the strong associations observed between H‐FVP variables and Phase 1a performance are unlikely to be measurement artifacts but reflect true mechanical determinants of COD execution.

From a practical perspective, these results highlight the need for practitioners to integrate both horizontal sprint profiling and phase‐specific COD assessments into athlete monitoring. While improvements in F0 and Pmax may enhance early COD execution, complementary interventions focusing on eccentric braking strength and unilateral re‐acceleration ability are required to optimize overall COD performance.

### 4.4 Practical Applications

The present findings indicate that horizontal sprint profiling can serve as a valuable monitoring tool in football, as improvements in F0 and Pmax may translate not only to linear sprinting but also to the early phases of COD performance. Optimizing the H‐FVP through individualized resisted sprint training may therefore enhance both sprint and COD ability.

At the same time, the limited correlations between H‐FVP and COD asymmetry suggest that sprint profiling alone cannot fully capture inter‐limb discrepancies. Practitioners are encouraged to integrate unilateral COD drills, eccentric strength training, and deceleration specific exercises to minimize asymmetries, which may improve COD efficiency and reduce injury risk. Furthermore, phase‐specific COD assessments can provide a more comprehensive understanding of directional change ability and help inform tailored training interventions.

### 4.5 Limitations

This study has several limitations that should be acknowledged. The relatively small sample size (*n* = 15) restricts statistical power and reduces the generalizability of the findings beyond elite U21 male football players. The use of the 1080 Sprint device enabled detailed profiling of sprint mechanics and COD specific phases, yet the outcomes are device‐specific and may not be directly comparable to studies employing alternative measurement systems. Moreover, the cross‐sectional design precludes causal inferences, and it remains uncertain whether modifications in H‐FVP would directly reduce inter‐limb asymmetries or enhance phase‐specific COD performance.

### 4.6 Future Research

Future studies should expand on these findings by including larger and more diverse cohorts of athletes to improve the generalizability of results. Longitudinal designs are warranted to determine whether targeted modifications of the H‐FVP can causally influence COD asymmetries and sub‐phase performance. Incorporating additional biomechanical measures, such as ground reaction forces, joint kinetics, or electromyography, would provide deeper insights into the mechanisms underlying phase‐specific COD performance. Finally, linking H‐FVP and COD phase characteristics to injury incidence over the course of a competitive season could clarify whether certain mechanical or asymmetry profiles represent modifiable risk factors in football players.

## 5 Conclusion

This study demonstrated that horizontal sprint mechanical properties, as described by the H‐FVP, were strongly related to overall COD and linear sprint performance, particularly through F0 and Pmax, while their correlations with inter‐limb asymmetries were limited. The most robust relationships emerged during early COD phases (Phase 1a and initial acceleration), whereas deceleration and re‐acceleration were less influenced by sprint mechanical outputs. These findings highlight the complementary value of combining horizontal sprint profiling with phase‐specific COD assessments to obtain a more complete understanding of an athlete's change of direction ability. From a practical perspective, optimizing the H‐FVP may enhance early COD performance, but reducing asymmetries and improving braking and re‐acceleration capacities require targeted, unilateral, and eccentric training strategies.

## Funding

This publication was prepared at Masaryk University as part of the project Doctoral Research in Kinanthropology IV ‐ MUNI/A/1583/2025, supported by the Specific University Research Grant provided by the Ministry of Education, Youth and Sports of the Czech Republic in 2025.

## Ethics Statement

The study was conducted in accordance with the Declaration of Helsinki. Data collection was carried out under a formal agreement between the Czech Football Federation and Masaryk University, with the primary purpose of academic reporting on athlete performance.

## Conflicts of Interest

The authors declare no conflicts of interest.

## Data Availability

Data supporting the findings of this study are stored at Masaryk University. Due to confidentiality agreements and ethical restrictions, raw data cannot be made publicly available. Processed datasets may be obtained from the corresponding author upon reasonable request.
